# Using intracameral cefuroxime as a prophylaxis for endophthalmitis

**Published:** 2008-03

**Authors:** David Yorston

**Affiliations:** Consultant Ophthalmologist, Tennent Institute of Ophthalmology, Gartnavel Hospital, 1053 Great Western Road, Glasgow G12 0YN, Scotland, UK.

**Figure:**
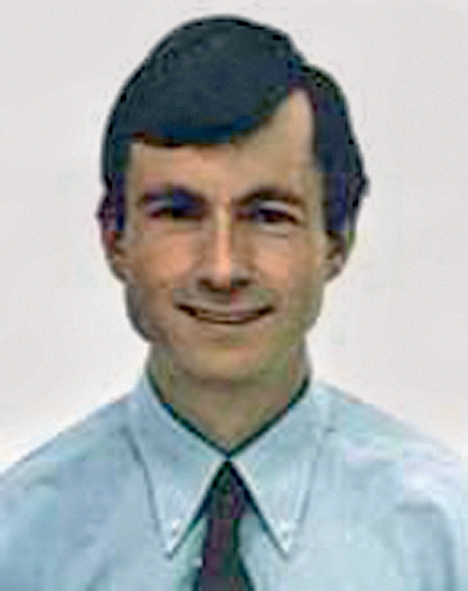


**Barry P, Seal DV, Gettinby G, Lees F, Peterson M, Revie CW; ESCRS Endophthalmitis Study Group**

**ESCRS study of prophylaxis of postoperative endophthalmitis after cataract surgery: preliminary report of principal results from a European multicenter study**

J Cataract Refract Surg 2006 Mar;32(3): 407–10

**Purpose:** To report results of the European Society of Cataract & Refractive Surgeons (ESCRS) multicentre study of the prophylaxis of endophthalmitis after cataract surgery.

**Setting:** Twenty-four ophthalmology units and eye clinics in Austria, Belgium, Germany, Italy, Poland, Portugal, Spain, Turkey, and the United Kingdom, with an administrative office in Ireland, a coordinating centre in England, and a data management and statistical unit in Scotland.

**Methods:** This partially masked randomised placebo-controlled multinational clinical study aimed to evaluate prospectively the prophylactic effect of an intracameral cefuroxime injection and/or perioperative levofloxacin eyedrops on the incidence of endophthalmitis after phacoemulsification cataract surgery. The study began in September 2003 and was terminated early in January 2006. The study used random allocation of patients in a 2x2 factorial design.

**Results:** By the end of 2005, complete follow-up records had been received for 13,698 study patients. Such a clear beneficial effect from the use of intracameral cefuroxime had been observed that it was agreed it would be unethical to continue the study and to wait for the completion of all follow-up procedures before reporting this important result. If total reported cases of endophthalmitis are considered, the incidence rate observed in those treatment groups **not** receiving cefuroxime prophylaxis (23 cases in 6,862 patients) was almost five times as high (odds ratio [OR] 4.59; 95% confidence interval [CI] 1.74–12.08; p=0.002) as that in the groups receiving this treatment (5 cases in 6,836 patients). If only cases proved to be due to infection are considered, the rate was more than five times as high (OR 5.32; 95% CI 1.55–18.26; p=0.008) in the treatment groups not receiving cefuroxime. Although the use of perioperative levofloxacin eyedrops as prophylaxis was also associated with a reduction in the observed incidence rate of postoperative endophthalmitis, this effect was smaller and was not statistically significant, whether total reported cases or only cases proven to be due to infection were used in calculating the rates. As not all follow-up procedures are complete, it is possible that further cases of endophthalmitis may be reported; however, it is not expected that this will alter the main conclusion. Nevertheless, it is anticipated that successful completion of follow-up procedures in all patients will increase the total number in the study to approximately 16,000.

**Conclusion:** Intracameral cefuroxime administered at the time of surgery significantly reduced the risk for developing endophthalmitis after cataract surgery.

## Comment

This paper presents the results of a large and well-designed randomised trial, which examined the effectiveness of an injection of cefuroxime 1 mg in the anterior chamber at the conclusion of cataract surgery. The results showed such a great benefit from the use of cefuroxime that the trial was stopped early, because it was considered unethical not to use the treatment.

From these figures, out of 10,000 cataract operations without postoperative intracameral cefuroxime, 23.3 would be expected to develop culture-positive endophthalmitis. With intracameral cefuroxime, this number would only be 4.4 (OR=5.32, 95%; CI 1.55–18.26). Globally, 10 million cataract operations are performed every year. This gives an incidence of endophthalmitis of 23,000 per year. If all surgeons used intracameral cefuroxime in every case, the incidence of endophthalmitis would be reduced to 4,400.

What about toxicity? It appears that the intracameral injection of cefuroxime is not toxic: Swedish researchers have published results on many thousands of eyes that have received intracameral cefuroxime without any adverse effects.^1^

It is possible that these results on the prophylactic effect of intracameral cefuroxime might be different in developing countries. This study was carried out in Europe, and it was also carried out on eyes that were having phacoemulsification. It is likely that the majority of these eyes had clear corneal incisions, with no sutures. Few clinics in developing countries routinely use phacoemulsification.They tend to favour extracapsular extraction, a technique in which the wound is covered with a conjunctival flap and which may be associated with a lower risk of infection. However, since the type of wound closure is not the only factor in the occurrance of endophthalmitis, similar prophylactic results may be obtained with the intracameral injection of cefuroxime at the end of extracapsular cataract extraction.

Despite these uncertainties, this study represents the best evidence we have regarding the prevention of this devastating complication.

**Preparing a cefuroxime injection**

**You will need the following:**

- Vial of 250 mg cefuroxime
- 2x10 ml normal saline
- 2 ml syringe
- 1 ml syringe

**Method**

1. Dissolve the cefuroxime in 12.5 ml of normal saline (20 mg/ml)
2. Draw up 1 ml of the cefuroxime solution (20 mg) in the 2 ml syringe
3. Make up to 2 ml with 1 ml normal saline (10 mg/ml)
4. Draw up 0.1 ml of this solution (1 mg) with the 1 ml syringe and inject into the anterior chamber, using a Rycroft cannula, through an anterior chamber paracentesis.
